# Multi-trait single-step genomic prediction accounting for heterogeneous (co)variances over the genome

**DOI:** 10.1038/s41437-019-0273-4

**Published:** 2019-10-22

**Authors:** Emre Karaman, Mogens S. Lund, Guosheng Su

**Affiliations:** Center for Quantitative Genetics and Genomics, Aarhus University, 8830 Tjele, Denmark

**Keywords:** Animal breeding, Genomics

## Abstract

Widely used genomic prediction models may not properly account for heterogeneous (co)variance structure across the genome. Models such as BayesA and BayesB assume locus-specific variance, which are highly influenced by the prior for (co)variance of single nucleotide polymorphism (SNP) effect, regardless of the size of data. Models such as BayesC or GBLUP assume a common (co)variance for a proportion (BayesC) or all (GBLUP) of the SNP effects. In this study, we propose a multi-trait Bayesian whole genome regression method (BayesN0), which is based on grouping a number of predefined SNPs to account for heterogeneous (co)variance structure across the genome. This model was also implemented in single-step Bayesian regression (ssBayesN0). For practical implementation, we considered multi-trait single-step SNPBLUP models, using (co)variance estimates from BayesN0 or ssBayesN0. Genotype data were simulated using haplotypes on first five chromosomes of 2200 Danish Holstein cattle, and phenotypes were simulated for two traits with heritabilities 0.1 or 0.4, assuming 200 quantitative trait loci (QTL). We compared prediction accuracy from different prediction models and different region sizes (one SNP, 100 SNPs, one chromosome or whole genome). In general, highest accuracies were obtained when 100 adjacent SNPs were grouped together. The ssBayesN0 improved accuracies over BayesN0, and using (co)variance estimates from ssBayesN0 generally yielded higher accuracies than using (co)variance estimates from BayesN0, for the 100 SNPs region size. Our results suggest that it could be a good strategy to estimate (co)variance components from ssBayesN0, and then to use those estimates in genomic prediction using multi-trait single-step SNPBLUP, in routine genomic evaluations.

## Background

Genomic selection was pioneered by the study of Meuwissen et al. (2001), and is rapidly becoming the state-of-the-art genetic selection methodology in many breeding programs around the world. The models proposed by Meuwissen et al. (2001) include a BLUP model, where the variances of single nucleotide polymorphism (SNP) effects are assumed to be the same for all SNPs (SNPBLUP), or specific to each SNP (BayesA and BayesB). Under a series of assumptions, the SNPBLUP model is equivalent to a mixed linear model, GBLUP (Habier et al. 2007), which uses a relationship matrix (**G**) computed from genetic markers (Nejati-Javaremi et al. 1997) to model covariances between individuals’ genetic effects (Stranden and Garrick 2009). This equivalency resulted in a widespread adoption of genomic prediction in genetic evaluations, because only an extra step of computation of **G** and its inverse is required for the traditional mixed model equations (Henderson 1984) used in animal breeding (Karaman et al. 2016). Moreover, it also allows all extensions of BLUP methodology, such as multiple-trait, random regression, or repeated measures to be easily implemented in genomic evaluations (Tiezzi and Maltecca 2015). The GBLUP model has been widely used to predict breeding values in animal species, such as cattle (Luan et al. 2009; Su et al. 2012b), pig (Lukić et al. 2015), sheep (Daetwyler et al. 2010a) and fish (Ødegård et al. 2014; Tsai et al. 2016), and accuracies from GBLUP were reported to be higher than those from traditional pedigree-based BLUP.

Although widely used in genomic evaluations, these BLUP-based genomic prediction models have some drawbacks. First, they ignore the fact that a large proportion of the SNPs may not have any influence on the trait of interest. Second, different loci or genomic regions may have rather different variances. The two models of Meuwissen et al. (2001), BayesA and BayesB, were proposed to overcome such drawbacks. Assuming SNP-specific variances, BayesA fits each of SNPs, while BayesB fits approximately 1-*π* of the SNPs, where *π* is the percentage of SNPs which have no influence on the trait of interest. When *π* = 0, BayesB is equivalent to BayesA. As pointed out by Gianola et al. (2009), both models are problematic as full conditional posteriors of the SNP-specific variances have only one additional degree of freedom compared to their priors regardless of the amount of data available. A simpler model that similarly fits approximately 1-*π* of the SNPs, but with a common variance, BayesC, was also proposed (Meuwissen 2009; Kizilkaya et al. 2010).

Zeng et al. (2016) introduced a Bayesian partitioned regression model for genomic prediction, which involves the selection of genome regions followed by the selection of SNPs within those selected regions. The model fits approximately 1 − Π of the regions assuming region-specific variances, and 1 − *π*_*s*_ of the SNPs within the region *s* assuming a common variance for the SNPs in the region. Referring to this “nested” variable selection structure of the model, it was termed as BayesN. The special case of the partitioned regression model of Zeng et al. (2016), i.e., BayesN with Π = *π*_*s*_ = 0 (hereafter, BayesN0), is equivalent to BayesA or GBLUP when a fixed region size is set at one SNP or the whole genome, respectively. We hypothesize that, at any other region size, but these two extreme sizes of genome regions, higher prediction accuracies can be obtained using BayesN0. Although it ignores the fact that a proportion of the genome regions, and therefore a proportion of the SNPs, may not have any influence on the trait of interest, prediction accuracy may increase compared to BayesA by benefiting from the increase in the accuracy in estimation of SNP variances, and compared to BLUP-based models by allowing SNPs in different regions to have different variances. Partitioning of the covariate matrix of marker genotypes, **M**, or in other words, assigning priors to genome regions rather than individual SNPs, was shown to influence the accuracy of genomic predictions (Brøndum et al. 2012; Gebreyesus et al. 2017; Karaman et al. 2018).

Many important traits in animal breeding have genetic correlations in varying sizes with one or more traits, and therefore, measurements of such correlated traits carry information for the genetic values of others. Several multi-trait models have been proposed for genomic prediction (Calus and Veerkamp 2011; Jia and Jannink 2012; Hayashi and Iwata 2013; Gebreyesus et al. 2017; Cheng et al. 2018b), and simulations have shown that genomic prediction accuracies from multi-trait models are superior to those from single-trait models (Calus and Veerkamp 2011; Jia and Jannink 2012; Guo et al. 2014; Karaman et al. 2018). Multi-trait genetic evaluation rely on the genetic association between the traits through the genetic variance and covariance structure. Models used for genomic prediction, therefore, should properly account for the makeup of these genetic (co)variance components to obtain the highest accuracy of prediction. When only a few genome regions explain a considerable amount of the variances and/or covariance in a two-trait analysis, models that account for the heterogeneous correlation structure over the genome may have advantages over the methods that assumes a constant correlation over the genome (Gebreyesus et al. 2017; Karaman et al. 2018).

The GBLUP model was extended to utilize all phenotypic, pedigree and genotypic information simultaneously, including phenotypic information on non-genotyped individuals, and termed as single-step GBLUP (ssGBLUP) (Christensen and Lund 2010; Aguilar et al. 2010). In ssGBLUP, the pedigree-based relationship matrix **A** and the genomic relationship matrix **G** are combined into a single matrix **H**. As for GBLUP, only an extra step for computation of **H** and its inverse is required for the traditional mixed model equations used in animal breeding (Misztal and Legarra 2017). However, ssGBLUP also suffers from the same drawbacks of GBLUP.

Fernando et al. (2014) proposed a class of single-step models, which not only unifies all available information as ssGBLUP does, but also accommodates any Bayesian whole genome regression model. This yields models of, for instance, ssBayesA or ssBayesN0, referring to the Bayesian whole genome regression model used in the single-step analysis. However, such an approach requires that all unknowns of the model to be estimated using Markov-chain Monte Carlo techniques which may be computationally infeasible especially in routine genomic evaluations. In genomic predictions using weighted GBLUP, it was shown that the use of the same SNP variances over a few years does not reduce prediction accuracy (Su et al. 2014). Indeed, in routine evaluations, variance components are not updated for each round of evaluation, because they are expected to be relatively consistent over time (Calus et al. 2014). An alternative to the fully Bayesian approach in Fernando et al. (2014) could be a strategy, where all necessary parameters are estimated using a Bayesian whole genome regression model first, and mixed model equations are then solved given the “known” values of the variance components, leading to a single-step SNPBLUP (ssSNPBLUP) model.

The aim of this study was three-fold: (i) to introduce a multi-trait whole genome regression model that allows heterogeneous (co)variances, (ii) to compare accuracies from single- and multi-trait genomic prediction, and (iii) to investigate the use of region-specific estimates of (co)variances in genomic predictions using ssSNPBLUP.

## Material and methods

### Data sets and simulations

The genotype data were simulated for five generations (Gen1−Gen5) based on real haplotypes of 2200 Holsteins (Gen0), as described in Karaman et al. (2018). At each generation, the number of males and females were kept constant at 200 and 2000, respectively, and the mating ratio was 1:10. Mating was completely at random, and selection was not considered. Each sire was mated twice with one of the ten dams to keep the population size at 2200 at each generation. Only the single nucleotide polymorphisms (SNPs) (11,154) located on first five chromosomes were considered.

Phenotypic values of the two traits were simulated to have heritabilities of 0.1 and 0.4, which represents low (L) and high (H) heritability traits, respectively. Total number of quantitative trait loci (QTL) was set at 200, which were randomly selected from the SNP set, ensuring that the average minor allele frequency (MAF) of QTL is 0.15 (Karaman et al. 2018). The criterion for the MAF of the QTL was based on the assumption that they in general have relatively low MAF (Goddard and Hayes 2009; Kemper and Goddard 2012). The QTL were randomly assigned into three groups according to their causal relationships with the traits. This was done by assuming a percentage of the total QTL (82%) had pleiotropic effects on two traits, while one half of the remaining QTL had effect on one trait, and one half on the other trait.

Two scenarios, G9 and N5, were considered in terms of the distribution of QTL effects and correlations for the effect of pleiotropic QTL. In the scenario G9, the effects of the pleiotropic QTL were achieved by simulating two correlated gamma variables (Dvorkin 2012) with marginal distributions of *G*(0.4, 1.66), and a correlation of 0.9. The 78% of those QTL were assigned to a correlation between effects on two traits of 0.9, and 22% of −0.9 randomly. The correlation group of −0.9 was achieved by switching the sign of QTL effect for one of the traits at random. The QTL effects, which were assumed to have a correlation of 0.9, were assigned a negative or positive sign at random for both traits. In the second scenario, scenario N5, effects of all pleiotropic QTL were simulated from a bivariate normal distribution with a correlation of 0.5. Although fluctuated across the replicates, all scenarios lead to genetic correlations of about 0.45 at Gen0. The QTL SNPs were excluded from the final data set of SNP for the analysis. Random residual effects were sampled from $$N\left({\mathbf{0}},\left[ {\begin{array}{*{20}{c}} {{\mathbf{I}}\sigma _{e_{\mathrm L}}^2} & {\mathbf{0}} \\ {\mathbf{0}} & {{\mathbf{I}}\sigma _{e_{\mathrm H}}^2} \end{array}} \right]\right)$$, where the sizes of $\sigma _{e_{\mathrm L}}^2$ and $\sigma _{e_{\mathrm H}}^2$ were determined according to heritabilities of 0.1 and 0.4, respectively.

Final data (see Table 1) were created by masking genotypes and/or phenotypes of the animals as follows. For generations 3 and 4, it was assumed that males had no phenotypes, but genotypes, while all females had phenotypes, and some fraction of them had also genotypes. Those genotyped females were selected completely at random. Generation 5 was used as validation population, where 500 randomly selected animals were assumed to be genotyped. Pedigree was traced back to Gen0. Animals had phenotypes on both traits, or none of them. In total, 20 replicates were generated.

**Table 1:** Number of animals with genotype and phenotype in each generation

Generation	G	G&P	P	Total
Gen0	–	–	–	2200
Gen1	–	–	–	2200
Gen2	–	–	–	2200
Gen3	200 (M)	500 (F)	1500 (F)	2200
Gen4	200 (M)	500 (F)	1500 (F)	2200
Gen5	500	–	–	2200

*G* genotype, *P* phenotype, *M* male, *F* female

### Models and methods

A novel multi-trait Bayesian whole genome regression model (BayesN0), single-step SNPBLUP and single-step Bayesian regression models introduced by Fernando et al. (2014) were compared for multi-trait genomic prediction. Single-trait analysis were also performed, but neither the models nor their theory were given in this paper, as the models are special cases of their multi-trait counterparts. In this section, we followed the notation in Fernando et al. (2014) as closely as possible.

#### Basic multi-trait model

A multi-trait mixed model including only general means as fixed effects and marker effects as random effects can be written as

1 $$\left[ {\begin{array}{*{20}{c}} {{\mathbf{y}}_{\mathrm L}} \\ {{\mathbf{y}}_{\mathrm H}} \end{array}} \right] = \left[ {\begin{array}{*{20}{c}} {{\mathbf{1}}_{\mathrm L}} & 0 \\ 0 & {{\mathbf{1}}_{\mathrm H}} \end{array}} \right]\left[ {\begin{array}{*{20}{c}} {\mu _{\mathrm L}} \\ {\mu _{\mathrm H}} \end{array}} \right] + \left[ {\begin{array}{*{20}{c}} {{\mathbf{M}}_{\mathrm L}} & 0 \\ 0 & {{\mathbf{M}}_{\mathrm H}} \end{array}} \right]\left[ {\begin{array}{*{20}{c}} {{\boldsymbol{\alpha }}_{\mathrm L}} \\ {{\boldsymbol{\alpha }}_{\mathrm H}} \end{array}} \right] + \left[ {\begin{array}{*{20}{c}} {{\mathbf{e}}_{\mathrm L}} \\ {{\mathbf{e}}_{\mathrm H}} \end{array}} \right],$$

where **y**_L_ and **y**_H_ are the vectors of phenotypes, **1** are vectors of ones, *μ*_L_ and *μ*_H_ are general means, **M**_L_ and **M**_H_ are the matrices of genotypes for *k* markers, ***α***_L_ and ***α***_H_ are the vectors of marker effects, and **e**_L_ and **e**_H_ are the vectors of random residual effects, for traits “L” and “H”, respectively. In our simulations, animals had records for both traits or none of them. Therefore, **M**_L_ = **M**_H_, and these matrices will be denoted as **M** hereinafter, to simplify the demonstration. Residuals, ${\mathbf{e}}\prime = \left[ {{\mathbf{e}}_{\mathrm L}^\prime ,{\mathbf{e}}_{\mathrm H}^\prime } \right]$, are typically assumed to follow a normal distribution, **e** | **R**_0_ ~ *N*(**0**, **R**_0_ ⊗ **I**), where $${\mathbf{R}}_0 = \left[ {\begin{array}{*{20}{c}} {\sigma _{e_{\mathrm L}}^2} & {\sigma _{e_{{\mathrm {LH}}}}} \\ {\sigma _{e_{{\mathrm {HL}}}}} & {\sigma _{e_{\mathrm H}}^2} \end{array}} \right]$$, and **I** is an identity matrix.

#### Multi-trait Bayesian partitioned regression (BayesN0)

The columns of **M** and vector ***α*** given in Eq. (1) can be divided into *S* subsets in a conformable manner:

$$\left[ {\begin{array}{*{20}{c}} {{\mathbf{y}}_{\mathrm L}} \\ {{\mathbf{y}}_{\mathrm H}} \end{array}} \right] = \left[ {\begin{array}{*{20}{c}} {{\mathbf{1}}_{\mathrm L}} & 0 \\ 0 & {{\mathbf{1}}_{\mathrm H}} \end{array}} \right]\left[ {\begin{array}{*{20}{c}} {\mu _{\mathrm L}} \\ {\mu _{\mathrm H}} \end{array}} \right] + \left[ {\begin{array}{*{20}{c}} {{\mathbf{M}}_1 \ldots {\mathbf{M}}_S} & 0 \\ 0 & {{\mathbf{M}}_1 \ldots {\mathbf{M}}_S} \end{array}} \right]\left[ {\begin{array}{*{20}{c}} {\begin{array}{*{20}{c}} {{\boldsymbol{\alpha }}_{{\mathrm L},1}} \\ \vdots \\ {{\boldsymbol{\alpha }}_{{\mathrm L},S}} \end{array}} \\ {\begin{array}{*{20}{c}} {{\boldsymbol{\alpha }}_{{\mathrm H},1}} \\ \vdots \\ {{\boldsymbol{\alpha }}_{{\mathrm H},S}} \end{array}} \end{array}} \right] + \left[ {\begin{array}{*{20}{c}} {{\mathbf{e}}_{\mathrm L}} \\ {{\mathbf{e}}_{\mathrm H}} \end{array}} \right],$$

where $${\mathbf{y}} = \left[ {\begin{array}{*{20}{c}} {{\mathbf{y}}_{\mathrm L}} \\ {{\mathbf{y}}_{\mathrm H}} \end{array}} \right]$$ involves the phenotypes of genotyped individuals only, **M**_1_, …, **M**_*S*_ are genotype matrices regarding genomic regions, and ***α***_*t*,1_, …, ***α***_*t*,*S*_ (*t* = L, H for low and high heritability traits, respectively) are vectors of SNP effects for corresponding genomic regions. We assume that all SNPs *j* (*j* = 1, …, *k*_*s*_) in the same genomic region *s* (*s* = 1, …, *S*) have the same (co)variance for the two traits:

$${\mathrm {var}}\left( {{\boldsymbol{\alpha }}_{sj}} \right) = {\mathrm {var}}\left[ {\begin{array}{*{20}{c}} {\alpha _{{\mathrm L},sj}} \\ {\alpha _{{\mathrm H},sj}} \end{array}} \right] = {\mathbf{B}}_s = \left[ {\begin{array}{*{20}{c}} {\sigma _{\alpha _{{\mathrm L},s}}^2} & {\sigma _{\alpha _{{\mathrm {LH}},s}}} \\ {\sigma _{\alpha _{{\mathrm {HL}},s}}} & {\sigma _{\alpha _{{\mathrm H},s}}^2} \end{array}} \right].$$

Likelihood of the model is given as:

$$\begin{array}{l}p({\mathbf{y}}\mid {\boldsymbol{\mu}},{\boldsymbol{\alpha}},{\mathbf{B}},{\mathbf{R}}) \propto \left| {\mathbf{R}} \right|^{ - \frac{1}{2}} {\exp} \left\{ { - \frac{1}{2}\left( {{\mathbf{y}} - {\mathbf{X}}{\boldsymbol{\mu}} - {\mathbf{M}}_{1}{\boldsymbol{\alpha}}_{1} - \ldots } \right.} \right.\\ \left. {\left. - {\mathbf{M}}_{S}{{\alpha}}_{S} \right)^\prime {\mathbf{R}}^{-1}({\mathbf{y}} - {\mathbf{X}}{\boldsymbol{\mu}} - {\mathbf{M}}_{1}{\boldsymbol{\alpha}}_{1} - \ldots - {\mathbf{M}}_{S}{{\alpha}}_{S})} \right\}\end{array}$$

where $${\mathbf{X}} = \left[ {\begin{array}{ll} {\mathbf{1}}_{\mathrm L} & {\mathbf{0}}\\ {\mathbf{0}} & {\mathbf{1}}_{\mathrm H} \end{array}} \right]$$, $$\boldsymbol{\mu} = \left[ \begin{array}{c} \mu _{\mathrm{L}} \\ \mu _{\mathrm{H}} \end{array} \right]$$, $${\mathbf{B}} = \left[ {\begin{array}{ll} {\mathbf{B}}_{\mathrm L} & {\mathbf{B}}_{{\mathrm {LH}}}\\ {\mathbf{B}}_{{\mathrm {HL}}} & {\mathbf{B}}_{\mathrm H} \end{array}} \right]$$ with ${\mathbf{B}}_i$ being diagonal matrices consisting of SNP variances (${\mathbf{B}}_{\mathrm L}$ and ${\mathbf{B}}_{\mathrm H}$) or covariances (${\mathbf{B}_{\mathrm {LH}}} = {\mathbf{B}_{\mathrm {HL}}}$), and $\mathbf{R} = \mathbf{R}_{0} \otimes \mathbf{I}$. The vector of fixed effects, ***μ***, were assigned a flat prior, and other parameters of the model were assigned a normal or an inverse Wishart (*IW*) prior for conjugacy:

$${\boldsymbol{\alpha}}_{sj}\mid {\mathbf{B}}_{s}\sim N(\mathbf{0},{\mathbf{B}}_{s})$$

$${\mathbf{e}}\mid {\mathbf{R}}_{0}\sim {\it N}({\mathbf{0}},{\mathbf{R}}_{0} \otimes \mathbf{I})$$

$${\mathbf{B}}_{s}\mid v_{B},{\mathbf{V}}_{B}\sim {IW}(v_{B},{\mathbf{V}}_{B})$$

$${\mathbf{R}}_{0}\mid v_{R},{\mathbf{V}}_{R}\sim {IW}(v_{R},{\mathbf{V}}_{R}).$$

Full conditional distributions of ***μ***, ***α***_*sj*_, **B**_*s*_, and **R**_0_ can be obtained after some algebra:

$$p\left({\boldsymbol{\mu}}\mid . \right)\sim N\left[ {\left({\mathbf{X}}\prime {\mathbf{R}}^{ - 1}{\mathbf{X}} \right)^{ - 1}{\mathbf{X}}\prime {\mathbf{R}}^{ - 1}{\mathbf{y}}^ {\ast} ,\left( {\mathbf{X}}\prime {\mathbf{R}}^{ - 1}{\mathbf{X}} \right)^{ - 1}} \right]$$

$$p\left({\boldsymbol{\alpha}}_{sj}\mid . \right)\sim N\left[ {\left({\mathbf{M}}_{j}^{ \ast \prime }{\mathbf{R}}^{ - 1}{\mathbf{M}}_{j}^{ \ast} + {\mathbf{B}}_{s}^{ - 1} \right)^{ - 1}{\mathbf{M}}_{j}^{ \ast \prime }{\mathbf{R}}^{ - 1}{\mathbf{y}}^ {\ast} , \left({\mathbf{M}}_{j}^{ \ast \prime }{\mathbf{R}}^{ - 1}{\mathbf{M}}_{j}^ {\ast} + {\mathbf{B}}_{s}^{ - 1} \right)^{ - 1}} \right]$$

$$p\left( {\mathbf{B}}_{s}\mid . \right)\sim {IW}\left[ v_{B} + k_{s},\left( {\mathbf{S}}_{B_{s}} + {\mathbf{V}}_{B} \right) \right]$$

$$p\left( {\mathbf{R}}_{0}\mid . \right)\sim {IW}\left[ v_{R} + n,\left( {\mathbf{S}}_{R} + {\mathbf{V}}_{R} \right) \right],$$

where, “.” stands for all other parameters and y^*^, y^*^ is the vector of phenotypes corrected for all other effects, $${\mathbf{M}}_j^ \ast = \left[ {\begin{array}{*{20}{c}} {{\mathbf{m}}_j} & {\mathbf{0}} \\ {\mathbf{0}} & {{\mathbf{m}}_j} \end{array}} \right]$$, ${\mathbf{S}}_{B_{s}} = \mathop {\sum}\nolimits_{j = 1}^{k_{s}} {\boldsymbol{\alpha}}_{sj} {\boldsymbol{\alpha} }_{sj}^{\prime}$ and ${\mathbf{S}}_{R} = \mathop {\sum}\nolimits_{i = 1}^{n} {\mathbf{e}}_{i} {\mathbf{e}}_{i}^{\prime}$. This multi-trait whole genome regression model was referred to as multi-trait BayesN0 throughout this paper, as it is an extension of a particular form of partitioned regression model (BayesN) introduced by Zeng et al. (2016), to multi-trait case. Note that when the size of region is fixed at one SNP or whole genome, model becomes equivalent to multi-trait BayesA or GBLUP, respectively.

#### Multi-trait single-step SNPBLUP

In the following expressions, *n* stands for the non-genotyped animals, and *g* stands for the genotyped animals. Note that in our simulations, animals had records for both traits or none of them. In a multi-trait single-step SNPBLUP (ssSNPBLUP) analysis, the phenotypes are modeled as (Fernando et al. 2014):

2 $${\mathbf{y}} = {\mathbf{X}}^ \ast {\boldsymbol{\mu }}^ \ast + {\mathbf{W}}{\boldsymbol{\alpha }} + {\mathbf{U}}{\boldsymbol{\epsilon }} + {\mathbf{e}}\,,$$

where $${\mathbf{y}} = \left[ {\begin{array}{*{20}{c}} {{\mathbf{y}}_{\mathrm L}} \\ {{\mathbf{y}}_{\mathrm H}} \end{array}} \right]$$ is the vector of phenotypes for genotyped and non-genotyped individuals, $${\boldsymbol{\mu }}^ \ast = \left[ {\begin{array}{*{20}{c}} {\mu _{\mathrm L}} \\ {\mu _{{\mathrm g},{\mathrm L}}} \\ {\mu _{\mathrm H}} \\ {\mu _{{\mathrm g},{\mathrm H}}} \end{array}} \right]$$*, μ*_L_ and *μ*_H_ are the overall means of the two traits*, μ*_g,L_ and *μ*_g,H_ are the differences between breeding values of genotyped and non-genotyped animals for the two traits, $${\mathbf{X}}^ \ast = \left[ {\begin{array}{*{20}{c}} {{\mathbf{X}}_{\mathrm L}^ \ast } & {\mathbf{0}} \\ {\mathbf{0}} & {{\mathbf{X}}_{\mathrm H}^ \ast } \end{array}} \right]$$ with $${\mathbf{X}}_{\mathrm L}^ \ast = {\mathbf{X}}_{\mathrm H}^ \ast = \left[ {\begin{array}{*{20}{c}} {\mathbf{1}} & { - {\mathbf{Z}}_{\mathrm n}{\mathbf{A}}_{{\mathrm {ng}}}{\mathbf{A}}_{{\mathrm {gg}}}^{ - 1}{\mathbf{1}}} \\ {\mathbf{1}} & { - {\mathbf{Z}}_{\mathrm g}{\mathbf{1}}} \end{array}} \right]$$, $${\mathbf{W}} = \left[ {\begin{array}{*{20}{c}} {{\mathbf{Z}}_{\mathrm L}} & {\mathbf{0}} \\ {\mathbf{0}} & {{\mathbf{Z}}_{\mathrm H}} \end{array}} \right]\left[ {\begin{array}{*{20}{c}} {{\mathbf{M}}_{\mathrm L}} & {\mathbf{0}} \\ {\mathbf{0}} & {{\mathbf{M}}_{\mathrm H}} \end{array}} \right]$$ with $${\mathbf{Z}}_{\mathrm L} = {\mathbf{Z}}_{\mathrm H} = \left[ {\begin{array}{*{20}{c}} {{\mathbf{Z}}_{\mathrm n}} & {\mathbf{0}} \\ {\mathbf{0}} & {{\mathbf{Z}}_{\mathrm g}} \end{array}} \right]$$ and $${\mathbf{M}}_{\mathrm L} = {\mathbf{M}}_{\mathrm H} = \left[ {\begin{array}{*{20}{c}} {\widehat {\mathbf{M}}_{\mathrm n}} \\ {{\mathbf{M}}_{\mathrm g}} \end{array}} \right]$$, $${\boldsymbol{\alpha }} = \left[ {\begin{array}{*{20}{c}} {{\boldsymbol{\alpha }}_{\mathrm L}} \\ {{\boldsymbol{\alpha }}_{\mathrm H}} \end{array}} \right]$$. **Z**_n_ and **Z**_g_ are incidence matrices relating breeding values of non-genotyped and genotyped animals to their phenotypes, ${\hat{\mathbf M}}_{\mathrm n}$ and **M**_g_ are matrices of imputed and observed genotypes for non-genotyped and genotyped animals, respectively, ***α***_L_ and ***α***_H_ are the vectors of allele substitution effects. The $${\mathbf{U}} = \left[ {\begin{array}{*{20}{c}} {{\mathbf{U}}_{\mathrm L}} & {\mathbf{0}} \\ {\mathbf{0}} & {{\mathbf{U}}_{\mathrm H}} \end{array}} \right]$$ with $${\mathbf{U}}_{\mathrm L} = {\mathbf{U}}_{\mathrm H} = \left[ {\begin{array}{*{20}{c}} {{\mathbf{Z}}_{\mathrm n}} \\ {\mathbf{0}} \end{array}} \right]$$ and $${\boldsymbol{\epsilon }} = \left[ {\begin{array}{*{20}{c}} {{\boldsymbol{\epsilon }}_{\mathrm L}} \\ {{\boldsymbol{\epsilon }}_{\mathrm H}} \end{array}} \right]$$, where ${\boldsymbol{\epsilon }}_{\mathrm L}$ and ${\boldsymbol{\epsilon }}_{{\mathrm H}}$ are the vectors of imputation residuals. The **e** is a vector of random residual effects assumed to follow **e** | **R**_0_ ~ *N*(**0**, **R**_0_ ⊗ **I**), where $${\mathbf{R}}_0 = \left[ {\begin{array}{*{20}{c}} {\sigma _{e_{\mathrm L}}^2} & {\sigma _{e_{{\mathrm {LH}}}}} \\ {\sigma _{e_{{\mathrm {HL}}}}} & {\sigma _{e_{\mathrm H}}^2} \end{array}} \right]$$, and **I** is an identity matrix. Vector of ***α*** is assumed to follow ***α*** | **B** ~ *N*(**0**, **B**) with $${\mathbf{B}} = \left[ {\begin{array}{*{20}{c}} {{\mathbf{B}}_{\mathrm L}} & {{\mathbf{B}}_{{\mathrm {LH}}}} \\ {{\mathbf{B}}_{{\mathrm {HL}}}} & {{\mathbf{B}}_{\mathrm H}} \end{array}} \right]$$, where **B**_*i*_ are diagonal matrices consisting of SNP variances (**B**_L_ and **B**_H_) or covariances (**B**_LH_ = **B**_HL_). Vector of ${\boldsymbol{\epsilon }}$ is assumed to follow ${\boldsymbol{\epsilon }}\mid {\mathbf{G}}_0,{\mathbf{A}}\sim N({\mathbf{0}},{\mathbf{G}}_0 \otimes {\mathbf{A}})$, where **G**_0_ is the additive genetic (co)variance matrix. The **A**_ng_, **A**_gg_ and **A**_nn_ are submatrices of the pedigree-based relationship matrix, **A**, corresponding to the relationships between non-genotyped and genotyped individuals, among the genotyped individuals, and among the non-genotyped individuals, respectively. The matrix of imputed genotypes, ${\hat{\mathbf M}}_{\mathrm n}$, is obtained with ${\mathbf{A}}_{{\mathrm {ng}}}{\mathbf{A}}_{{\mathrm {gg}}}^{ - 1}{\mathbf{M}}_{\mathrm g}$ (Fernando et al. 2014).

The mixed model equations corresponding to the model in Eq. (2) is as follows.

3 $$\left[ {\begin{array}{*{20}{c}} {{\mathbf{X}}^{ \ast \prime }{\mathbf{R}}^{ - 1}{\mathbf{X}}^ \ast } & {{\mathbf{X}}^{ \ast \prime }{\mathbf{R}}^{ - 1}{\mathbf{W}}} & {{\mathbf{X}}_{\mathrm n}^{ \ast \prime }{\mathbf{R}}^{ - 1}{\mathbf{U}}_{\mathrm n}} \\ {{\mathbf{W}}^\prime {\mathbf{R}}^{ - 1}{\mathbf{X}}^ \ast } & {{\mathbf{W}}^\prime {\mathbf{R}}^{ - 1}{\mathbf{W}} + {\mathbf{B}}^{ - {\mathbf{1}}}} & {{\mathbf{W}}_{\mathrm n}^\prime {\mathbf{R}}^{ - 1}{\mathbf{U}}_{\mathrm n}} \\ {{\mathbf{U}}_{\mathrm n}^\prime {\mathbf{R}}^{ - 1}{\mathbf{X}}^ \ast } & {{\mathbf{U}}_{\mathrm n}^\prime {\mathbf{R}}^{ - 1}{\mathbf{W}}} & {{\mathbf{U}}_{\mathrm n}^\prime {\mathbf{R}}^{ - 1}{\mathbf{U}}_{\mathrm n} + {\mathbf{G}}_0^{ - 1} \otimes {\mathbf{A}}^{ - {\mathrm {nn}}}} \end{array}} \right]\left[ {\begin{array}{*{20}{c}} {\widehat {\boldsymbol{\mu }}} \\ {\widehat {\boldsymbol{\alpha }}} \\ {\widehat {\boldsymbol{\epsilon }}} \end{array}} \right] = \left[ {\begin{array}{*{20}{c}} {{\mathbf{X}}^{ \ast \prime }{\mathbf{R}}^{ - 1}{\mathbf{y}}} \\ {{\mathbf{W}}^\prime {\mathbf{R}}^{ - 1}{\mathbf{y}}} \\ {{\mathbf{U}}_{\mathrm n}^\prime {\mathbf{R}}^{ - 1}{\mathbf{y}}} \end{array}} \right]$$

The **A**^−nn^ is the part of the inverse of pedigree-based relationship matrix, **A**, corresponding to the non-genotyped individuals, and **R** = **R**_0_ ⊗ **I**.

#### Multi-trait single-step BayesN0 (ssBayesN0)

The single-step SNPBLUP requires the estimation of (co)variance components, and then use of these in mixed model equations to estimate breeding values. In contrast, Bayesian approach can be used to obtain the vector of fixed and random effect estimates, $\left[ {\widehat {\boldsymbol{\mu }},\widehat {\boldsymbol{\alpha }},\widehat {\boldsymbol{\epsilon }}} \right]^\prime$, the genetic and residual variance components, and the SNP (co)variances simultaneously, as in the original paper of Fernando et al. (2014). In principle, any Bayesian whole genome regression model can be incorporated in this single-step model, and BayesN0 was used here (ssBayesN0). Likelihood of the ssBayesN0 model is given as:

$$\begin{array}{l}p({\mathbf{y}}\mid {\boldsymbol{\mu }}^ \ast ,{\boldsymbol{\alpha }},{\mathbf{B}},{\mathbf{R}}) \propto \left| {\mathbf{R}} \right|^{ - \frac{1}{2}}\,\exp\,\left\{ { - \frac{1}{2}({\mathbf{y}} - {\mathbf{X}}^ \ast {\boldsymbol{\mu }}^ \ast - {\mathbf{W}}_1{\boldsymbol{\alpha }}_1 - \ldots - {\mathbf{W}}_S{\boldsymbol{\alpha }}_S - {\mathbf{U}}{\boldsymbol{\epsilon }})^\prime {\mathbf{R}}^{ - 1}} \right.\\ \left. {({\mathbf{y}} - {\mathbf{X}}^ \ast {\boldsymbol{\mu }}^ \ast - {\mathbf{W}}_1{\boldsymbol{\alpha }}_1 - \cdots - {\mathbf{W}}_S{\boldsymbol{\alpha }}_S - {\mathbf{U}}{\boldsymbol{\epsilon }})} \right\},\end{array}$$

where matrices and parameters are as specified earlier. A flat prior was assumed for μ*. Priors for ***α***, **e**, **B**_*s*_ and **R**_0_ were the same as in BayesN0. A multivariate normal prior, ${\boldsymbol{\epsilon }}\mid {\mathbf{G}}_0,{\mathbf{A}}\sim N({\mathbf{0}},{\mathbf{G}}_0 \otimes {\mathbf{A}})$, was assumed for the vector of ${\boldsymbol{\epsilon }}$, and **G**_0_ was assigned an inverse Wishart prior, **G**_0_ | *v*_G_, **V**_G_ ~ *IW*(*v*_G_, **V**_G_). Full conditional distributions of μ^*^, ***α***_*sj*_, **B**_*s*_, ${\boldsymbol{\epsilon }}$, **G**_0_, **R**_0_ can be obtained after some algebra:

$$p\left( {{\boldsymbol{\mu }}^ \ast \mid .} \right)\sim N\left[ {\left( {{\mathbf{X}}^{ \star \prime }{\mathbf{R}}^{ - 1}{\mathbf{X}}^ \ast } \right)^{ - 1}{\mathbf{X}}^\prime {\mathbf{R}}^{ - 1}{\mathbf{y}}^ \ast ,{\mkern 1mu} \left( {{\mathbf{X}}^{ \star \prime }{\mathbf{R}}^{ - 1}{\mathbf{X}}^ \ast } \right)^{ - 1}} \right]$$

$$p\left( {{\boldsymbol{\alpha }}_{sj}\mid .} \right)\sim N\left[ {\left( {{\mathbf{W}}_j^{ \ast \prime }{\mathbf{R}}^{ - 1}{\mathbf{W}}_j^ \ast + {\mathbf{B}}_s^{ - 1}} \right)^{ - 1}{\mathbf{W}}_j^{ \ast \prime }{\mathbf{R}}^{ - 1}{\mathbf{y}}^ \ast ,{\mkern 1mu} \left( {{\mathbf{W}}_j^{ \ast \prime }{\mathbf{R}}^{ - 1}{\mathbf{W}}_j^ \ast + {\mathbf{B}}_s^{ - 1}} \right)^{ - 1}} \right]$$

$$p\left( {{\mathbf{B}}_s\mid .} \right)\sim IW\left[ {v_B + k_s,\left( {{\mathbf{S}}_{B_s} + {\mathbf{V}}_B} \right)} \right]$$

$$p\left( {{\boldsymbol{\epsilon }}|.} \right)\sim N\left[ {\left( {{\mathbf{U}}_{\mathrm n}^\prime {\mathbf{R}}^{ - 1}{\mathbf{U}}_{\mathrm n} + {\mathbf{G}}_0^{ - 1} \otimes {\mathbf{A}}^{ - {\mathrm {nn}}}} \right)^{ - 1}{\mathbf{U}}_{\mathrm n}^\prime {\mathbf{R}}^{ - 1}{\mathbf{y}}^ \ast ,{\mkern 1mu} \left( {{\mathbf{U}}_{\mathrm n}^\prime {\mathbf{R}}^{ - 1}{\mathbf{U}}_{\mathrm n} + {\mathbf{G}}_0^{ - 1} \otimes {\mathbf{A}}^{ - {\mathrm {nn}}}} \right)^{ - 1}} \right]$$

$$p\left( {{\mathbf{G}}_0\mid .} \right)\sim IW\left[ {v_G + N_{\mathrm n},\left( {{\mathbf{S}}_G + {\mathbf{V}}_G} \right)} \right]$$

$$p\left( {{\mathbf{R}}_0\mid .} \right)\sim IW\left[ {v_R + n,\left( {{\mathbf{S}}_R + {\mathbf{V}}_R} \right)} \right]$$

where y^*^, ${\textbf{S}}_{B_{S}}$ and **S**_*R*_ are as defined before, $${\mathbf{W}}_j^ \ast = \left[ {\begin{array}{*{20}{c}} {{\mathbf{w}}_j} & {\mathbf{0}} \\ {\mathbf{0}} & {{\mathbf{w}}_j} \end{array}} \right]$$, *N*_n_ is the number of non-genotyped individuals, and $${\mathbf{S}}_G = \left[ {\begin{array}{*{20}{c}} {{\boldsymbol{\epsilon }}_{\mathrm L}^\prime {\mathbf{A}}^{ - {\mathrm {nn}}}{\boldsymbol{\epsilon }}_{\mathrm L}} & {{\boldsymbol{\epsilon }}_{\mathrm L}^\prime {\mathbf{A}}^{ - {\mathrm {nn}}}{\boldsymbol{\epsilon }}_{\mathrm H}} \\ {{\boldsymbol{\epsilon }}_{\mathrm H}^\prime {\mathbf{A}}^{ - {\mathrm {nn}}}{\boldsymbol{\epsilon }}_{\mathrm L}} & {{\boldsymbol{\epsilon }}_{\mathrm H}^\prime {\mathbf{A}}^{ - {\mathrm {nn}}}{\boldsymbol{\epsilon }}_{\mathrm H}} \end{array}} \right]$$.

### Statistical analysis

Single- and multi-trait models of BayesN0 and single-step BayesN0 (ssBayesN0) were fitted with varying region sizes (one SNP, 100 SNPs, a whole chromosome and the whole genome). The parameters of the priors for SNP, residual and genetic (co) variance matrices in the multi-trait models were ${\mathbf{V}}_B = (v_B - 2 - 1)\widetilde {\mathbf{B}}$ where $\widetilde {\mathbf{B}} = \frac{{\widetilde {\mathbf{G}}_0}}{{{\sum} 2 p_j\left( {1 - p_j} \right)}}$, ${\mathbf{V}}_R = \left( {v_R - 2 - 1} \right)\widetilde {\mathbf{R}}_0$, and ${\mathbf{V}}_G = \left( {v_G - 2 - 1} \right)\widetilde {\mathbf{G}}_0$, which were derived from the mean of an inverse Wishart distributed random variable, and *v*_*B*_ = *v*_*R*_ = *v*_*G*_ = 5. It is worth noting that inverse Wishart distribution imply a scaled inverse chi-square distribution for each variance with specific parameters (Wang et al. 2018). That is, e.g., ${\mathbf{B}}_{s_{11}} = \sigma _{\alpha _{{\mathrm L},s}}^2\sim \chi ^{ - 2}\left( {4,\frac{{\tilde \sigma _{\alpha _{{\mathrm L},s}}^2}}{2}} \right)$, where $\tilde \sigma _{\alpha _{{\mathrm L},s}}^2$ is the first diagonal element in $\widetilde {\mathbf{B}}$.

Single-trait BayesN0 and ssBayesN0 models were special cases of their multi-trait counterparts, for which the multivariate normal priors for SNP effects, model residuals and imputation residuals were replaced with univariate normal priors $\left( {{\mathrm{e}}.{\mathrm{g}}.,\:\alpha _{{\mathrm L},sj}\sim N\left( {0,\sigma _{\alpha _{{\mathrm L},s}}^2} \right)} \right)$, and inverse Wishart priors for the (co)variance components were replaced with scaled inverted chi-square priors $\left( {{\mathrm{e}}.{\mathrm{g}}.,\:\sigma _{\alpha _{{\mathrm L},s}}^2\sim \chi ^{ - 2}\left( {{\mathrm {df}},S_{\mathrm L}^2} \right)} \right)$, for conjugacy. Parameters for these scaled inverted chi-square prior distributions for SNP, residual and genetic variances were df = 4 and a scale parameter, derived from the expected value of a scaled inverse chi-square distributed random variable $\left( {{\mathrm{e}}.{\mathrm{g}}.,\:S_{\mathrm L}^2 = \frac{{\tilde \sigma _{\alpha _{{\mathrm L},s}}^2\left( {{\mathrm {df}} - 2} \right)}}{{{\mathrm {df}}}},\:{\mathrm{where}}\;\:\tilde \sigma _{\alpha _{{\mathrm L},s}}^2 = \frac{{\tilde \sigma _{g_{\mathrm L}}^2}}{{{\sum} 2 p_j\left( {1 - p_j} \right)}}} \right)$ (Habier et al. 2010a). That is, e.g., $\sigma _{\alpha _{{\mathrm L},s}}^2\sim \chi ^{ - 2}\left( {4,\frac{{\tilde \sigma _{\alpha _{{\mathrm L},s}}^2}}{2}} \right)$. Hence, not only the mean, but also the distribution of priors for the variances were consistent between the single- and multi-trait analysis, with only difference being the value of variance components used. The matrices of $\widetilde {\mathbf{G}}_0$ and $\widetilde {\mathbf{R}}_0$ used in priors for multi-trait analysis, and genetic $\left( {\tilde \sigma _g^2} \right)$ and residual variances $\left( {\tilde \sigma _e^2} \right)$ used in priors for single-trait analysis, were the estimates obtained by fitting single or multi-trait Ridge-Regression models at SNP level, respectively, using the JWAS (Cheng et al. 2018a) package in Julia (Bezanson et al. 2017).

Markov-chain Monte Carlo (MCMC) algorithm with Gibbs sampling method was used to obtain samples of each parameter from its full conditional posterior distribution. Chain length for the analyses using BayesN0 and ssBayesN0 consisted of 50,000 or 70,000 cycles, of which the first 30,000 or 50,000 cycles were discarded as burn-in, respectively. Convergence was tested by comparing results for the two chain lengths (50,000 vs. 70,000) on a random subset of the replicates and region sizes (Zeng et al. 2018). Every tenth sample of the post burn-in cycles were stored for posterior analysis, yielding 2,000 posterior samples. Mean value of the posterior samples was used as the estimate of each parameter. The change in accuracy of prediction was negligible for 70,000 compared to 50,000 cycles of Markov chain, and therefore, the results from the chain length of 50,000 were presented.

For single- and multi-trait ssSNPBLUP models, the genetic and residual (co)variances and SNP (co)variances were obtained as the mean values of the posterior samples from BayesN0 or ssBayesN0. The genetic (co)variances required in mixed model equations for $\hat {\boldsymbol{\epsilon}}$ were computed as the mean of the (co)variances of the breeding values at each MCMC cycle for BayesN0, or directly as the mean of genetic (co)variances for ssBayesN0. Hereafter, analysis using the variance components from BayesN0 and ssBayesN0 will be referred to as ssSNPB1 and ssSNPB2, respectively. The ssSNPB1 and ssSNPB2 models were solved with the Conjugate Gradients method with diagonal preconditioning using the IterativeSolvers package in Julia, and convergence tolerance was chosen to be 10^−12^. All analyses were performed using self-written scripts in Julia.

The predicted breeding values of animals using multi-trait BayesN0 were obtained from

$$\widehat {\mathbf{g}}_t = {\mathbf{M}}\widehat {\boldsymbol{\alpha }}_t,\quad t = \text{L,H}.$$

The predicted breeding values of animals using single-step models, ssBayesN0, ssSNPB1 and ssSNPB2, were obtained from:

4 $$\widehat {\mathbf{g}}_t = \left[ {\begin{array}{*{20}{c}} { - {\mathbf{A}}_{{\mathrm {ng}}}{\mathbf{A}}_{{\mathrm {gg}}}^{ - 1}{\mathbf{1}}} \\ { - {\mathbf{1}}} \end{array}} \right]\hat \mu _{{\mathrm g},t} + \left[ {\begin{array}{*{20}{c}} {\widehat {\mathbf{M}}_{\mathrm n}} \\ {{\mathbf{M}}_{\mathrm g}} \end{array}} \right]\widehat {\boldsymbol{\alpha }}_t + \left[ {\begin{array}{*{20}{c}} {{\mathbf{Z}}_{\mathrm n}} \\ {\mathbf{0}} \end{array}} \right]\widehat {\boldsymbol{\epsilon }}_t,\quad t = \text{L,H}$$

Prediction accuracy was assessed as the correlation between true and predicted breeding values of validation individuals. The bias of prediction was assessed based on the slope of the regression of true breeding values on the estimated breeding values of validation individuals. Accuracy for single- and multi-trait models with different region sizes were compared for each trait, and each model separately. Prediction accuracy for all methods was compared for each trait and at each scenario of region size. All comparisons were performed separately for genotyped and non-genotyped individuals using a two-sided paired *t*-tests, for which accuracies were paired across each replicate for the same validation population. A Bonferroni correction was used to control the Type 1 error rate of 0.05, caused by multiple comparisons.

## Results

### Bayesian whole genome regression (BayesN0)

Prediction accuracies from single- and multi-trait BayesN0 models are given in Tables 2 and 4 for genotyped individuals in validation population, at varying sizes of genome region. Grouping 100 adjacent SNPs generally provided the highest accuracies for both single- and multi-trait models, with some exceptions in scenario N5. Accuracies for different region sizes were generally ranked as 100 SNPs > 1 SNP > 1 Chr > WG in scenario G9. When a multi-trait model was used in scenario G9, prediction accuracy for the region size of 100 SNPs were about 4 and 12 percentage points higher for low heritability trait (L), and about 3 and 8 percentage points higher for high heritability trait (H), compared to those for region sizes of one SNP (BayesA) and whole genome (GBLUP), respectively. Using multi-trait BayesN0 with a region size of 100 SNPs resulted in higher accuracies than corresponding single-trait BayesN0 for both traits, though not always significant. Bias for predicting breeding values of genotyped individuals is shown in Supplementary Tables S1 and S3. Regression coefficients were generally closer to 1 for trait H in both scenarios. They were higher than 1 particularly for single-trait analysis of trait L in scenario G9 and single- and multi-trait analysis of trait L in scenario N5.

**Table 2:** Accuracies for genotyped individuals using single- and multi-trait models in scenario G9

Trait^1^	Region size^2^	Single-trait^3^				Multi-trait			
		BayesN0	ssSNPB1	ssBayesN0	ssSNPB2	BayesN0	ssSNPB1	ssBayesN0	ssSNPB2
L^4^	1 SNP	_ab_0.349^e^	_ab_0.452^cd^	_ab_0.470^d^	_ab_0.478^c^	_b_0.437^d^	_b_0.536^b^	_b_0.554^b^	_a_0.574^a^
	100 SNPs	_a_0.365^d^	_a_0.460^c^	_a_0.478^c^	_a_0.479^c^	_a_0.481^c^	_a_0.559^b^	_a_0.590^a^	_a_0.590^a^
	1 Chr	_b_0.335^c^	_b_0.434^b^	_bc_0.444^b^	_bc_0.445^b^	_b_0.402^b^	_c_0.493^a^	_c_0.497^a^	_b_0.499^a^
	WG	_b_0.335^d^	_b_0.433^b^	_c_0.433^bc^	_c_0.433^b^	_c_0.362^cd^	_d_0.461^ab^	_d_0.472^a^	_c_0.473^a^
H	1 SNP	_b_0.587^g^	_b_0.683^e^	_b_0.689^de^	_b_0.700^bc^	_b_0.593^f^	_b_0.688^cd^	_b_0.699^b^	_a_0.712^a^
	100 SNPs	_a_0.611^f^	_a_0.698^d^	_a_0.716^b^	_a_0.716^b^	_a_0.622^e^	_a_0.707^c^	_a_0.725^a^	_a_0.725^a^
	1 Chr	_c_0.552^e^	_c_0.650^bd^	_c_0.651^cd^	_c_0.651^abcd^	_c_0.558^e^	_c_0.655^ac^	_c_0.657^ab^	_b_0.657^ab^
	WG	_d_0.538^d^	_c_0.642^c^	_c_0.642^bc^	_c_0.643^bc^	_d_0.543^d^	_d_0.644^abc^	_d_0.646^ab^	_c_0.646^a^

^1^L and H: low (0.1) and high (0.4) heritability traits, respectively

^2^Chr chromosome, WG whole genome

^3^ssSNPB1 and ssSNPB2: Single-step SNPBLUP, for which the variance components were obtained from BayesN0 and ssBayesN0, respectively

^4^Different alphabets mean significantly different values at a Type 1 error rate of 0.05 with Bonferroni correction. Subscripts and superscripts stand for comparisons within column and row, respectively, for each trait

### Single-step genomic prediction

Prediction accuracies from single- and multi-trait analysis are given in Tables 2–5. Similar to BayesN0, accuracies for different region sizes were generally ranked as 100 SNPs > 1 SNP > 1 Chr > WG in scenario G9. For single-trait analysis of trait L, accuracies from the region size of 1 SNP and/or WG were similar to, or even slightly higher than, that of region size of 100 SNPs in scenario N5. Using ssSNPB1 improved accuracies for genotyped individuals compared to using BayesN0, for both single- and multi-trait analysis. Accuracies from ssBayesN0 were generally similar to or somewhat higher than those from ssSNPB1, particularly in scenario G9. Using single-step SNPBLUP with (co)variances obtained from ssBayesN0, i.e., ssSNPB2, yielded similar accuracies to the corresponding ssBayesN0 model. Accuracies from ssSNPB2 were similar to, though sometimes slightly higher in scenario G9, those from ssSNPB1 for non-genotyped animals. For non-genotyped animals, taking 100 adjacent SNPs as a genome region provided similar to or slightly higher accuracies than taking one SNP as a genome region, but higher accuracies than taking whole genome as a genome region, in scenario G9. For scenario N5, on the other hand, all region sizes generally lead to similar accuracies for non-genotyped animals. Regression coefficients were generally closer to 1 for trait H, but higher than 1 for trait L in scenario N5 (Supplementary Tables S1–S4).

**Table 3:** Accuracies for non-genotyped individuals using single- and multi-trait models in scenario G9

Trait^1^	Region size^2^	Single-trait^3^			Multi-trait		
		ssSNPB1	ssBayesN0	ssSNPB2	ssSNPB1	ssBayesN0	ssSNPB2
L^4^	1 SNP	_ab_0.351^c^	_ab_0.357^c^	_ab_0.361^c^	_b_0.402^b^	_b_0.406^b^	_a_0.418^a^
	100 SNPs	_a_0.355^c^	_a_0.363^c^	_a_0.363^c^	_a_0.412^b^	_a_0.426^a^	_a_0.427^a^
	1 Chr	_b_0.340^b^	_b_0.342^b^	_bc_0.342^b^	_c_0.378^a^	_c_0.377^a^	_b_0.378^a^
	WG	_b_0.337^c^	_b_0.337^c^	_c_0.336^c^	_d_0.361^abc^	_d_0.365^b^	_c_0.366^a^
H	1 SNP	_a_0.526^cd^	_a_0.528^d^	_a_0.531^bc^	_a_0.529^bcd^	_a_0.533^b^	_a_0.537^a^
	100 SNPs	_a_0.530^c^	_a_0.535^b^	_a_0.535^b^	_a_0.534^b^	_a_0.539^a^	_a_0.539^a^
	1 Chr	_b_0.513^abc^	_b_0.513^c^	_b_0.513^bc^	_b_0.516^abc^	_b_0.517^ab^	_b_0.517^a^
	WG	_b_0.511^bc^	_b_0.511^c^	_b_0.511^bc^	_b_0.513^abc^	_b_0.514^ab^	_c_0.514^a^

^1^L and H: low (0.1) and high (0.4) heritability traits, respectively

^2^Chr chromosome, WG whole genome

^3^ssSNPB1 and ssSNPB2: Single-step SNPBLUP, for which the variance components were obtained from BayesN0 and ssBayesN0, respectively

^4^Different alphabets mean significantly different values at a Type 1 error rate of 0.05 with Bonferroni correction. Subscripts and superscripts stand for comparisons within column and row, respectively, for each trait

**Table 4:** Accuracies for genotyped individuals using single- and multi-trait models in scenario N5

Trait^1^	Region size^2^	Single-trait^3^				Multi-trait			
		BayesN0	ssSNPB1	ssBayesN0	ssSNPB2	BayesN0	ssSNPB1	ssBayesN0	ssSNPB2
L^4^	1 SNP	_a_0.314^d^	_a_0.432^b^	_a_0.432^b^	_a_0.433^b^	_a_0.362^c^	_a_0.470^a^	_a_0.469^a^	_a_0.470^a^
	100 SNPs	_a_0.313^e^	_a_0.428^bc^	_a_0.434^b^	_ab_0.429^c^	_a_0.367^d^	_ab_0.468^a^	_a_0.475^a^	_a_0.474^a^
	1 Chr	_a_0.309^d^	_b_0.419^c^	_b_0.420^c^	_b_0.419^c^	_b_0.341^d^	_c_0.447^abc^	_b_0.447^b^	_b_0.450^a^
	WG	_a_0.314^c^	_a_0.431^ab^	_ab_0.430^b^	_a_0.432^a^	_b_0.342^c^	_bc_0.447^ab^	_ab_0.459^ab^	_ab_0.460^ab^
H	1 SNP	_b_0.545^d^	_a_0.651^c^	_b_0.654^bc^	_a_0.658^ab^	_b_0.548^d^	_a_0.654^bc^	_b_0.657^b^	_a_0.662^a^
	100 SNPs	_a_0.554^d^	_a_0.654^c^	_a_0.663^a^	_a_0.662^ab^	_a_0.559^d^	_a_0.657^bc^	_a_0.666^a^	_a_0.665^a^
	1 Chr	_c_0.537^b^	_b_0.642^a^	_c_0.645^a^	_b_0.645^a^	_c_0.540^b^	_b_0.644^a^	_c_0.646^a^	_b_0.647^a^
	WG	_c_0.537^b^	_b_0.644^a^	_c_0.645^a^	_b_0.646^a^	_c_0.539^b^	_b_0.645^a^	_c_0.648^a^	_b_0.648^a^

^1^L and H: low (0.1) and high (0.4) heritability traits, respectively

^2^Chr chromosome, WG whole genome

^3^ssSNPB1 and ssSNPB2: Single-step SNPBLUP, for which the variance components were obtained from BayesN0 and ssBayesN0, respectively

^4^Different alphabets mean significantly different values at a Type 1 error rate of 0.05 with Bonferroni correction. Subscripts and superscripts stand for comparisons within column and row, respectively, for each trait

**Table 5:** Accuracies for non-genotyped individuals using single- and multi-trait models in scenario N5

Trait^1^	Region size^2^	Single-trait^3^			Multi-trait		
		ssSNPB1	ssBayesN0	ssSNPB2	ssSNPB1	ssBayesN0	ssSNPB2
L^4^	1 SNP	_a_0.328^c^	_a_0.325^c^	_a_0.326^c^	_a_0.357^a^	_a_0.353^b^	_a_0.355^ab^
	100 SNPs	_a_0.327^b^	_a_0.327^b^	_a_0.326^b^	_a_0.357^a^	_a_0.355^a^	_a_0.355^a^
	1 Chr	_a_0.324^c^	_a_0.325^c^	_a_0.324^c^	_ab_0.349^ab^	_a_0.347^b^	_a_0.350^a^
	WG	_a_0.327^b^	_a_0.325^b^	_a_0.327^ab^	_b_0.343^ab^	_a_0.350^b^	_a_0.352^a^
H	1 SNP	_a_0.506^d^	_b_0.507^cd^	_a_0.510^b^	_a_0.508^bc^	_ab_0.509^b^	_a_0.512^a^
	100 SNPs	_a_0.507^c^	_a_0.511^ab^	_a_0.511^ab^	_a_0.509^bc^	_a_0.512^ab^	_a_0.512^a^
	1 Chr	_ab_0.503^b^	_bc_0.504^ab^	_b_0.504^ab^	_ab_0.505^a^	_bc_0.505^ab^	_b_0.506^ab^
	WG	_b_0.503^b^	_c_0.503^b^	_b_0.503^b^	_b_0.504^ab^	_c_0.506^a^	_b_0.506^a^

^1^L and H: low (0.1) and high (0.4) heritability traits, respectively

^2^Chr chromosome, WG whole genome

^3^ssSNPB1 and ssSNPB2: Single-step SNPBLUP, for which the variance components were obtained from BayesN0 and ssBayesN0, respectively

^4^Different alphabets mean significantly different values at a Type 1 error rate of 0.05 with Bonferroni correction. Subscripts and superscripts stand for comparisons within column and row, respectively, for each trait

## Discussion

### Single- vs. multi-trait genomic prediction

Multi-trait analysis generally led to higher accuracies than their single-trait counterparts for trait L (*h*^2^ = 0.1), and similar to or higher accuracies than their single-trait counterparts for trait H (*h*^2^ = 0.4) (Tables 2–5). This was expected because the gain of accuracy from multi-trait over single-trait genomic prediction is more profound for low heritability traits that are genetically correlated with a high heritability trait (Jia and Jannink 2012; Guo et al. 2014). Hayashi and Iwata (2013) compared accuracies from single- and multi-trait analysis for traits with a genetic correlation of 0.7, and reported that accuracy for a low heritability trait (*h*^2^ = 0.1) was improved with multi-trait analysis, while accuracy for a high heritability (*h*^2^ = 0.8) trait remained unchanged. For a low heritability (*h*^2^ = 0.05) trait, which had incomplete data, Guo et al. (2014) showed that accuracy of genomic prediction was improved when a genetically correlated (*r*_g_ = 0.5) trait with high heritability (*h*^2^ = 0.3) was available. Cheng et al. (2018b) reported that the mean of the posterior probability that a marker has a null effect was higher (0.97 vs. 0.74) in multi-trait analysis (BayesCΠ) compared to single-trait analysis (BayesC*π*) for gall volume (*h*^2^ = 0.12), when the correlated trait was presence (or absence) of rust (*h*^2^ = 0.21), in Loblolly Pine (*Pinus taeda* L.) (Resende et al. 2012).

Beside heritability, another factor influencing accuracy is the absolute difference between genetic and residual correlations (Schaeffer 1984; Thompson and Meyer 1986). In this study, the simulated residual correlation was null and the genetic correlation was moderate (0.45), though the estimates of those correlations varied around the simulated true values. Averaged over the replicates, genetic correlations were generally overestimated, whereas the residual correlations were nearly zero and varied only after second decimal, in both scenarios and for all region sizes. Genetic correlations were 0.47 and 0.45 from BayesN0 with the region size of 100 SNPs, and 0.56 and 0.51 from GBLUP (BayesN0 with whole genome as one region), for scenarios G9 and N5, respectively (results not given elsewhere). Those were 0.49 and 0.47 for ssBayesN0 with the region size of 100 SNPs, and 0.54 and 0.48 for ssGBLUP (ssBayesN0 with whole genome as one region), for scenarios G9 and N5, respectively (results not given elsewhere). These small deviations of genetic correlations from their true values are expected to have little influence in variance of prediction error (PEV), and multi-trait models can increase the precision of breeding value estimates by reducing PEV compared to single-trait models (Schaeffer 1984). The PEV was additionally computed for BayesN0 and ssBayesN0, from the variance of posterior samples for breeding values of genotyped individuals in validation population. Averaged over region sizes, the mean reduction in PEV from multi-trait BayesN0 were about 2.5% for trait L and 0.5% for trait H, and 5% for trait L and 0.5% for trait H, in scenarios G9 and N5, respectively (results not given elsewhere). The mean reduction in PEV from multi-trait ssBayesN0 were about 9% for trait L and 0.9% for trait H, and 6% for trait L and 0.8% for trait H, in scenarios G9 and N5, respectively (results not given elsewhere). Bias for single-trait analysis was relatively high for trait L particularly in scenario G9 (Supplementary Tables S1–S4), however, it was generally reduced by using multi-trait models.

In multi-trait genomic prediction, correlation structures between the traits is central to gaining advantage in prediction accuracy over single-trait predictions (Gebreyesus et al. 2017). Our results showed that the improvement from multi-trait analysis over single-trait analysis were dependent on whether the genetic makeup of the (co)variance structure of the studied traits (Tables 2–5) were accounted for, and this will be discussed in detail in the later sections.

### Accounting for heterogeneous (co)variances across the genome using BayesN0

Multi-trait genomic prediction rely on the genetic association between the traits through the genetic variances and covariances, which may vary across the genome. A few genome regions may explain a substantial proportion of the covariance, whereas others account for nearly no covariance between the traits (Sørensen et al. 2012). Moreover, covariances between particular traits may be positive for some regions and negative for others, while the overall genetic correlations are low/high (Li et al. 2017; Gebreyesus et al. 2017). This study investigated the affect of assigning priors to genome regions, which were defined as fixed number of SNPs (one SNP, 100 SNPs, one chromosome or whole genome), on accuracy in multi-trait genomic prediction.

Genomic prediction rests on the LD between QTL and SNPs (Meuwissen et al. 2001). Although the simulation settings in this study resulted in correlations of QTL effects that fall into different categories, it may be of a general question where does the heterogenity of (co)variances over the genome come from, or what does it refer to. It can be shown that the best linear predictor of SNP effects is ${\boldsymbol{\alpha }}_t = {\mathbf{V}}_{\mathrm M}^{ - 1}{\mathbf{V}}_{{\mathrm {MQ}}}{\boldsymbol{\gamma }}_t$ (*t* = L, H), where ***γ***_*t*_ is the vector of QTL effects, **V**_M_ is the (co)variance matrix of SNP genotypes, and **V**_MQ_ is the covariance matrix of SNP and QTL genotypes (de los Campos et al. 2015). Note that for a QTL that affect only L (or H), corresponding row of ***γ***_H_ (or ***γ***_L_) is zero. Under some assumptions, (co)variance of the SNP effects are proportional to ${\mathbf{V}}_{{\mathrm M}_s{\mathrm Q}_s}{\mathbf{V}}_{{\mathrm M}_s{\mathrm Q}_s}^\prime$, for genome region *s* (*s* = 1, …, *S*). Because recombination rates vary over the genome, and SNPs are typically in imperfect LD with QTL, each ${\textrm{{V}}_{M_{s}}Q_{s}}$ may be different (Wang et al. 2013), resulting in genome having a different (co)variance pattern at the SNP level than that of at the QTL level (de los Campos et al. 2015).

Multi-trait BayesA (BayesN0 with region size of one SNP) was able to account for the heterogeneous correlation structure across the genome to some extent, compared to multi-trait GBLUP (BayesN0 with whole genome as one region), which assumes a constant correlation across the genome (Tables 2 and 4). Accuracies were further improved when a group of 100 SNPs were allowed to have a common (co)variance. It should be noted that the choice of region sizes was arbitrary, and therefore, the region size of 100 SNPs may not be optimal. Alternatively, regions can be achieved by grouping SNPs based on fixed length of genomic region or LD information. Because the extent of LD is highly variable in different populations (Wang et al. 2013), and varies with respect to SNP density (Goddard and Hayes 2009), the decision of optimal region size is crucial to obtain highest accuracy of genomic prediction (Gebreyesus et al. 2017).

Simulation studies have shown that Bayesian whole genome regression models, which allow variances of SNP effects differing among loci or genome regions, perform better than GBLUP model (Meuwissen et al. 2001; Lund et al. 2009; Karaman et al. 2018). In real-data applications, the accuracy of genomic prediction using the Bayesian whole genome regression models led to similar to or higher accuracies than methods assuming a constant variance structure (e.g., GBLUP) across the genome (Hayes et al. 2009; Habier et al. 2010b; Su et al. 2012a). The benefit from Bayesian whole genome regression models was larger for traits with simple genetic architectures (Coster et al. 2010; Daetwyler et al. 2010b; Clark et al. 2011; Karaman et al. 2018). Examples for such traits can be milk protein composition traits, in which a substantial proportion of the variance is explained by a few QTL (Heck et al. 2009; Schopen et al. 2011). Gebreyesus et al. (2017) reported that BaysesAS model resulted in higher prediction reliabilities than GBLUP for milk protein composition traits, when 100 SNPs were assumed to have a common (co)variance, based on a data set from 50 K SNP panel in Danish Holstein cattle.

For prediction of traits with large effect QTL, the GBLUP model, in which a selection of SNPs, i.e., SNPs identified in earlier genome-wide association studies (GWAS) or identified via GWAS using the current data (*de novo* GWAS, Spindel et al. (2016)), are considered as fixed effects, can provide accuracies as high as those from Bayesian whole genome prediction methods (Spindel et al. 2016; Lopes et al. 2017). Although the approach is relatively straightforward, it either requires a priori information about the SNPs for the traits of interest, or running a GWAS prior to genomic prediction. Depending on the choice of statistical method, the definition of the QTL region and the significance threshold, different sets of SNPs can be achieved even with the same data, and QTL regions that explain a substantial proportion of the variance may also not always be identified for all traits (Goddard et al. 2016; Lopes et al. 2017).

By applying BayesN0, one has the possibility of putting emphasis on genome regions with large effect, without requiring any prior knowledge on the QTL region affecting the trait(s), or without running a *de novo* GWAS (Lopes et al. 2017). For practical application in breeding programs, we think this is an advantage over GBLUP, in which “some” SNPs are considered as fixed effects. Our results for scenario N5 imply that the advantage of grouping SNPs in BayesN0 over GBLUP is not limited only to traits with a few QTL with large effect and many with small effects (scenario G9). In scenario N5, BayesN0 with 100 SNPs region size led to a similar accuracy to that from GBLUP in single-trait analysis of trait L, but to a higher accuracy than GBLUP in multi-trait analysis of trait L. Since using a multi-trait model may be beneficial for traits by increasing the amount of information, it can be argued that the accuracies from single-trait analysis of trait L would also differ among the region sizes, for the intermediate sizes of data (Karaman et al. 2016). For asymptotically large sizes of data, on the other hand, there might be little or no benefit of using more sophisticated methods compared to GBLUP (Karaman et al. 2016; Cheng et al. 2018b).

In a simulation study for single-trait genomic prediction, Zeng et al. (2018) showed that BayesN was superior to BayesB when the QTL had relatively low MAF, for a panel consisting of 50 K SNPs. It is, however, unclear if this was due to selection of regions at each cycle of MCMC, or due to reliable estimation of SNP variances by assuming common variance to SNPs in each region, rather than assuming a variance specific to each SNP. In that study, fitting ten SNPs per region also provided higher accuracies of prediction than fitting two SNPs per region. Hess et al. (2017) further allowed SNPs within a region to have different variances, in a study using 50 K SNP panel of an admixed cattle population in New Zealand. There was no advantage of BayesN over BayesB, for milk fat yield, live-weight and somatic cell score. They also showed that fitting all SNPs in a region resulted in slightly higher accuracies than fitting only two SNPs per region.

### Accounting for heterogeneous (co)variances across the genome using single-step Bayesian regression

Implementation of our novel Bayesian multi-trait model (ssBayesN0) using the methodology of Fernando et al. (2014) yielded accuracies for genotyped individuals in the range of 0.47–0.59 and 0.45–0.48 for trait L, and 0.65–0.73 and 0.65–0.67 for trait H, in scenarios G9 and N5, respectively (Tables 2 and 4). In a single-step analysis using Bayesian regression (Fernando et al. 2014), taking one SNP as a genome region is equivalent to single-step BayesA (ssBayesA) and taking whole genome as one region is equivalent to single-step GBLUP (ssGBLUP). Our results indicate that ssBayesA can lead to higher accuracies than ssGBLUP in a multi-trait analysis, by exploiting the heterogeneous (co)variance structure across the genome. However, similar to the regular BayesA (Meuwissen et al. 2001), the information in the data that is utilized by ssBayesA is limited, due to its strong dependency on the prior for (co)variance of SNP effects (Gianola et al. 2009). This dependency on the prior was overcome to some extent by assuming a common (co)variance for 100 adjacent SNPs using ssBayesN0, which generally led to higher accuracies than ssBayesA and ssGBLUP for both trait L and H (Tables 2 and 4). Similarly, prediction accuracy for non-genotyped individuals were increased about 6 and 0.5 percentage points for trait L, and about 2.5 and 0.6 percentage points for trait H, for scenarios G9 and N5, respectively, when region size was changed from whole genome to 100 SNPs in multi-trait analyses (Tables 3 and 5).

For genotyped individuals, using multi-trait ssBayesN0 led to higher accuracies than using multi-trait BayesN0 (Tables 2 and 4). As other Bayesian whole genome regression models, BayesN0 can only use the phenotypes of genotyped animals. The ssBayesN0, on the other hand, simultaneously uses the phenotypes of genotyped animals (1000) and non-genotyped animals (3000, the genotypes were imputed) (Table 1) to estimate the SNP effects in the MCMC procedure, while accounting for the error in imputation for non-genotyped individuals with phenotypes. This enhances the data size used in estimation of SNP effects, which has a key role to obtain reliable prediction of breeding values (Daetwyler et al. 2008; Goddard 2009; Karaman et al. 2016; Cheng et al. 2018b).

### Practical implementation of single-step models using previously estimated (co)variance components

In this study, the estimates of the (co)variances were obtained from BayesN0 or ssBayesN0. The former led to ssSNPB1 model for which the (co)variance components were obtained using only the information of genotyped individuals, while the latter led to ssSNPB2 model for which the (co)variance components were obtained using the information of genotyped and non-genotyped individuals. In practice, the (co)variance components can be estimated less frequently compared to routine genomic evaluations without harming the prediction accuracies (Su et al. 2014).

For genotyped individuals, ssSNPB1 model yielded higher accuracies than BayesN0, at all region sizes in multi-trait analysis (Tables 2 and 4). This was due to more accurate estimation of SNP effects by the use of phenotypes of non-genotyped individuals. The ssBayesN0 and ssSNPB2, where the (co)variance components from ssBayesN0 were used, generally yielded similar accuracies. This was not surprising, because similar to BayesC0 and SNPBLUP being equivalent models, ssBayesN0 and ssSNPB2, are also equivalent. The ssSNPB2 generally led to higher accuracies than ssSNPB1 in scenario G9, and similar to or slightly higher accuracies than ssSNPB1 in scenario N5, in multi-trait analyses.

Accuracies for non-genotyped animals were generally similar among the models, i.e., ssSNPB1, ssBayesN0 and ssSNPB2, in multi-trait analysis. Analysis using the model of Fernando et al. (2014) starts with an explicit imputation of markers for non-genotyped individuals, using pedigree information and genotypes of genotyped relatives. Then, marker effects and imputation residuals (***ϵ***) accounting for the part of breeding values, which cannot be modeled by imputed markers, are estimated (Gao et al. 2018). Imputation residual is added to marker-based breeding value (sum of individual SNP effects) of non-genotyped individuals to obtain their total breeding values (Fernando et al. 2014). The breeding value of genotyped individuals, on the other hand, is composed only of sum of individual SNP effects. Hence, a change in the accuracy of SNP effect estimates has less impact on the accuracy of breeding value estimates for non-genotyped individuals than for genotyped individuals (Zhou et al. 2018).

One way to account for heterogeneous (co)variance structure in single-step genomic prediction could be to construct weighted **G** matrices (Zhang et al. 2010), and in turn their weighted **H** matrix counterparts (Fragomeni et al. 2017) for ssGBLUP. In an earlier study (Karaman et al. 2018), we have shown that weighted multi-trait GBLUP can reach accuracies similar to that of the Bayesian whole genome regression model which was used to derive weights. This was expected, because those “weighted” relationship matrices are indeed implicit to Bayesian whole genome regression methods (Fernando and Gianola 2018; Karaman et al. 2018). A drawback of the approach using weighted relationship matrices is that it requires the computation of a number of relationship matrices which increase with the number of traits in a multi-trait model, and not only the computing time but also the storage of such **H** matrices might be impractical for genomic prediction using weighted ssGBLUP in routine evaluations. Moreover, compared to ssGBLUP, the equations needed to be solved for ssSNPBLUP does not grow with the number of genotyped individuals, and the inverse of the combined relationship matrix, **H**, is not needed (Fernando et al. 2014).

We did not focus on the computational (dis)advantages of ssSNPBLUP, nor its convergency properties. Both ssGBLUP and ssSNPBLUP, though, are known to have some computational challenges (Taskinen et al. 2017). Averaged over the scenarios and region sizes, ssSNPB1 and ssSNPB2 models achieved relative convergence of 10^−12^ in 200 and 203 iterations (average of two traits) in single-trait analysis, and in 435 and 478 iterations in multi-trait analysis, respectively. It should be noted that these numbers apply only to the current data, and could vary with equivalent formulations of the models (Taskinen et al. 2017) or with a preconditioner other than diagonal used in this study.

## Conclusions

In this study, a multi-trait whole genome regression model, BayesN0, was proposed. The model has its equivalent counterparts when the region size is set at one SNP (BayesA) or the whole genome (GBLUP). Our results showed that assigning priors to genome regions defined as fixed number of SNPs, e.g., 100 SNPs, may improve accuracies over BayesA and GBLUP by accounting for heterogeneous (co)variance structure across the genome efficiently. The model was also implemented in single-step (ssBayesN0) Bayesian regression approach, which unifies pedigree, phenotypes and genotypes in a single analysis. Highest prediction accuracies were obtained when 100 adjacent SNPs were assumed to have a common (co)variance in ssBayesN0. For routine genomic evaluations, it could be a good strategy to estimate (co)variance components from ssBayesN0, and then to use those estimates in genomic prediction using multi-trait single-step SNPBLUP. Such a strategy has the potential to provide reliable estimates of breeding values for both genotyped and nongenotyped individuals.

## Supplementary information

Supplementary tables

## Data Availability

Genotype and pedigree data can be found at 10.5061/dryad.v4126t4, along with a file including necessary SNP information (chromosome ID and base-pair position). The data and the methodology described previously are sufficient to reproduce the results of this study.
