# Correction: Multi-trait single-step genomic prediction accounting for heterogeneous (co)variances over the genome

**DOI:** 10.1038/s41437-020-0299-7

**Published:** 2020-02-21

**Authors:** Emre Karaman, Mogens S. Lund, Guosheng Su

**Affiliations:** Center for Quantitative Genetics and Genomics, Aarhus University, 8830 Tjele, Denmark

**Keywords:** Animal breeding, Genomics

**Correction to: Heredity**

10.1038/s41437-019-0273-4

In the original PDF version of this article, the below equation was incorrectly displayed.

Instead of:

$${\text{where}}\,{\mathbf{X}} = \left[ {{\mathbf{1}}_{\mathrm{L}}{\mathbf{001}}_{\mathrm{H}}} \right],{\boldsymbol{\mu }} = \left[ {\begin{array}{*{20}{l}} {\mu _{\mathrm{L}}} \hfill \\ {\mu _{\mathrm{H}}} \hfill \end{array}} \right],{\mathbf{B}} = \left[ {{\mathbf{B}}_{\mathrm{L}}{\mathbf{B}}_{{\mathrm{LH}}}{\mathbf{B}}_{{\mathrm{HL}}}{\mathbf{B}}_{\mathrm{H}}} \right]$$

The equation should be displayed as follows:

$${\text{where}}\,{\mathbf{X}} = \left[ {\begin{array}{*{20}{l}} {{\mathbf{1}}_{\mathrm{L}}} \hfill & {\mathbf{0}} \hfill \\ {\mathbf{0}} \hfill & {{\mathbf{1}}_{\mathrm{H}}} \hfill \end{array}} \right],{\boldsymbol{\mu }} = \left[ {\begin{array}{*{20}{l}} {\mu _{\mathrm{L}}} \hfill \\ {\mu _{\mathrm{H}}} \hfill \end{array}} \right],{\mathbf{B}} = \left[ {\begin{array}{*{20}{l}} {{\mathbf{B}}_{\mathrm{L}}} & {{\mathbf{B}}_{{\mathrm{LH}}}} \\ {{\mathbf{B}}_{{\mathrm{HL}}}} & {{\mathbf{B}}_{\mathrm{H}}} \end{array}} \right]$$

This error has been corrected in the PDF version of the article. The HTML version of the article was correct at the time of publication.

