# Supplementary material for: Multi-trait single-step genomic prediction accounting for heterogeneous (co)variances over the genome
Source: Heredity (Edinb). 2019 Oct 22;124(2):274–87. doi: 10.1038/s41437-019-0273-4 (PMC6972913; doi:10.1038/s41437-019-0273-4)
Supplement: Supplementary file 1 — Supplementary tables [file 41437_2019_273_MOESM1_ESM.pdf]

Table S1: Regression of true breeding values on estimated breeding values for genotyped individuals in scenario G9

Trait[1]	Region size[2]	Single-Trait[3]				Multi-Trait			
		BayesN0	ssSNPB1	ssBayesN0	ssSNPB2	BayesN0	ssSNPB1	ssBayesN0	ssSNPB2
L	1 SNP	1.184	1.078	1.127	1.061	1.110	1.035	1.065	0.967
	100 SNPs	1.189	1.048	1.093	0.972	1.158	1.036	1.076	0.975
	1 Chr	1.230	1.127	1.069	1.029	1.138	1.090	1.038	1.004
	WG	1.365	1.228	1.024	1.013	1.095	1.093	1.017	1.006
H	1 SNP	0.967	0.955	1.001	0.944	0.962	0.948	0.995	0.933
	100 SNPs	0.978	0.967	1.008	0.962	0.977	0.961	1.000	0.950
	1 Chr	1.014	1.017	0.997	0.987	1.016	1.018	1.000	0.986
	WG	0.991	1.012	0.989	0.988	0.999	1.016	0.990	0.988

[1]*L* and *H*: low (0.1) and high (0.4) heritability traits, respectively.

[2]Chr: chromosome; WG: Whole genome.

[3]ssSNPB1 and ssSNPB2: Single-step SNPBLUP, for which the variance components were obtained from BayesN0 and ssBayesN0, respectively.

Table S2: Regression of true breeding values on estimated breeding values for non-genotyped individuals in scenario G9

Trait[1]	Region size[2]	Single-Trait[3]			Multi-Trait		
		ssSNPB1	ssBayesN0	ssSNPB2	ssSNPB1	ssBayesN0	ssSNPB2
L	1 SNP	1.147	1.149	1.089	1.068	1.076	1.001
	100 SNPs	1.127	1.130	1.035	1.063	1.078	0.998
	1 Chr	1.211	1.111	1.078	1.117	1.039	1.013
	WG	1.294	1.083	1.070	1.130	1.032	1.023
H	1 SNP	0.997	1.027	0.983	0.989	1.022	0.974
	100 SNPs	0.999	1.019	0.988	0.991	1.012	0.977
	1 Chr	1.071	1.038	1.032	1.070	1.041	1.032
	WG	1.060	1.031	1.031	1.063	1.033	1.031

[1]*L* and *H*: low (0.1) and high (0.4) heritability traits, respectively.

[2]Chr: chromosome; WG: Whole genome.

[3]ssSNPB1 and ssSNPB2: Single-step SNPBLUP, for which the variance components were obtained from BayesN0 and ssBayesN0, respectively.

Table S3: Regression of true breeding values on estimated breeding values for genotyped individuals in scenario N5

Trait[1]	Region size[2]	Single-Trait[3]				Multi-Trait			
		BayesN0	ssSNPB1	ssBayesN0	ssSNPB2	BayesN0	ssSNPB1	ssBayesN0	ssSNPB2
L	1 SNP	1.354	1.320	1.409	1.348	1.206	1.185	1.249	1.141
	100 SNPs	1.362	1.277	1.374	1.219	1.209	1.136	1.183	1.046
	1 Chr	1.494	1.372	1.283	1.215	1.224	1.227	1.168	1.123
	WG	1.662	1.538	1.374	1.357	1.522	1.490	1.181	1.166
H	1 SNP	1.006	0.980	1.024	0.960	1.004	0.977	1.024	0.955
	100 SNPs	1.009	0.969	1.004	0.947	1.011	0.967	1.004	0.940
	1 Chr	1.031	1.017	0.984	0.974	1.024	1.017	0.993	0.980
	WG	1.006	1.013	0.986	0.986	1.009	1.017	0.985	0.983

[1]*L* and *H*: low (0.1) and high (0.4) heritability traits, respectively.

[2]Chr: chromosome; WG: Whole genome.

[3]ssSNPB1 and ssSNPB2: Single-step SNPBLUP, for which the variance components were obtained from BayesN0 and ssBayesN0, respectively.

Table S4: Regression of true breeding values on estimated breeding values for non-genotyped individuals in scenario N5

Trait[1]	Region size[2]	Single-Trait[3]			Multi-Trait		
		ssSNPB1	ssBayesN0	ssSNPB2	ssSNPB1	ssBayesN0	ssSNPB2
L	1 SNP	1.343	1.404	1.353	1.212	1.245	1.167
	100 SNPs	1.319	1.365	1.252	1.189	1.179	1.074
	1 Chr	1.434	1.321	1.267	1.312	1.198	1.164
	WG	1.568	1.343	1.332	1.536	1.186	1.176
H	1 SNP	1.024	1.047	1.005	1.021	1.048	1.000
	100 SNPs	1.029	1.042	1.002	1.027	1.040	0.997
	1 Chr	1.074	1.024	1.018	1.074	1.033	1.025
	WG	1.052	1.017	1.017	1.055	1.015	1.013

[1]*L* and *H*: low (0.1) and high (0.4) heritability traits, respectively.

[2]Chr: chromosome; WG: Whole genome.

[3]ssSNPB1 and ssSNPB2: Single-step SNPBLUP, for which the variance components were obtained from BayesN0 and ssBayesN0, respectively.
